# Link between Parkinson’s disease and melanoma: insights into the influence of the *PARK* gene family

**DOI:** 10.3389/fonc.2025.1506744

**Published:** 2025-08-11

**Authors:** Jinghua Wu, Haojun Xiong, Jinhua Chen, Dengrong Yang, Yujing Li, Jinglai Wang, Jiaoyu Chen, Ruixia Zhang, Ruiqi Zhang, Xiwei Li, Feng Li, Runnan Zhang, Zhi Yang

**Affiliations:** ^1^ Department of Dermatology, First Affiliated Hospital of Kunming Medical University, Kunming, China; ^2^ Department of Dermatology, The Affiliated Hospital, Southwest Medical University, Luzhou, China; ^3^ School of Pharmacy, Kunming Medical University, Kunming, China

**Keywords:** Parkinson’s disease, PARK gene family, α-synuclein, pathogenesis, melanoma

## Abstract

Parkinson’s disease (PD) is a common neurodegenerative disorder characterized by damage to dopaminergic neurons within the substantia nigra region of the midbrain. Melanoma, on the other hand, is a malignant skin tumor formed by the abnormal proliferation of melanocytes, often linked to genetic predisposition and ultraviolet exposure. Emerging evidence confirms a significant association between PD and melanoma, with individuals afflicted with PD displaying a higher susceptibility to melanoma development. The *PARK* family genes, known for their involvement in PD etiology, emerge as key players in elucidating this intricate relationship. Through a comprehensive review, it becomes evident that different *PARK* gene mutations exert varied impacts on both PD and melanoma pathogenesis. For instance, mutations in *PARK1/4* influence α-synuclein aggregation in both PD and melanoma, while *PARK8* mutations modulate autophagy pathways in both PD and melanoma. The roles of *PARK2* and *PARK13* in melanoma warrant further investigation. Additionally, *PARK6* mutations influence mitophagy mechanisms in PD and melanoma, with implications regarding melanoma proliferation through the PI3K/AKT pathway. Therefore, delineating the precise contributions of *PARK* genes to PD and melanoma pathophysiology holds paramount importance in devising therapeutic strategies for both PD and melanoma.

## 1 Introduction

Parkinson’s disease (PD) is currently the second most prevalent neurodegenerative disease, and is usually accompanied by metabolic abnormalities (1, 2). Central to its pathology is the aberrant aggregation of α-synuclein (α-syn), which is implicated to a variety of neurodegenerative conditions (3). Alongside its hallmark, PD manifests numerous non-motor symptoms in addition to motor symptoms such as autonomic dysfunction, olfactory impairment (4), sleep disturbances (5), and cognitive decline (6). Skin manifestations in PD, often overlooked due to their non-specific nature and the lack of objective clinical measures, encompass symptoms such as dryness, pruritus, erythema, and desquamation, especially affecting the scalp and face (7).

There is increasing evidence suggesting a link between PD and various dermatological conditions such as melanoma (8), seborrheic dermatitis (9), dysregulated sweating (10), and bullous pemphigoid (11). Despite fundamental differences - PD entails cell degeneration while melanoma leads to cell proliferation - epidemiological data reveals a higher risk of melanoma among PD patients (12), with reciprocal risks noted in melanoma patients developing PD. A previous study reported that over a 5-year period, the risk of developing melanoma in patients with PD was 2.4-fold higher than in the healthy population (13). Melanoma, a highly malignant melanocyte-derived tumor, underscores the neuroprotective role of neuromelanin through dopaquinone scavenging (8), whereas, patients with PD exhibit significantly lower neuromelanin levels (14). Previous studies suggest that levodopa, a cornerstone PD therapy, may contribute to the development of melanoma, due to shared dopamine and melanin biosynthetic pathways (15), although contradictory findings exist (16).

The discovery of the *PARK* gene has played a crucial role in the history of PD research. *PARK1* or *PARK4* was the first *PARK* gene discovered to cause PD in 1996 (17). This discovery sets the stage for subsequent research. Then, *PARK1-PARK18* genes were identified as being associated with PD (18). Inzelberg et al. reported that 48% of melanoma tissue samples have mutations in at least one *PARK* gene and 25% have mutations in multiple *PARK* genes (19). The high proportion of mutations in *PARK* genes in melanoma suggests a possible correlation between melanoma and PD.

Mutations within the *PARK* gene family are strongly associated with PD (20), yet their implications in melanoma remain unmapped. For instance, *PARK1/4* encoded α-syn (*PARK1/4*) influences melanin and neuromelanin biosynthesis by regulation of tyrosinase (Tyr), tyrosine hydroxylase (TH), and peroxidase (21). Elucidating shared pathogenic mechanisms in PD and melanoma holds significant therapeutic options for patients with PD and melanoma. However, the precise mechanisms underlying their association remains enigmatic. This review aims to dissect the roles of *PARK* genes - *PARK1/4*, *PARK2*, *PARK5*, *PARK6*, *PARK7*, *PARK8*, *PARK13*, *PARK14*, and *PARK18* in both PD and melanoma, thereby fostering novel therapeutic strategies for these debilitating conditions.

## 2 Role of *PARK* family in PD and melanoma

### 2.1 α-syn/*PARK1* a high risk for melanomas

**Figure 1:**
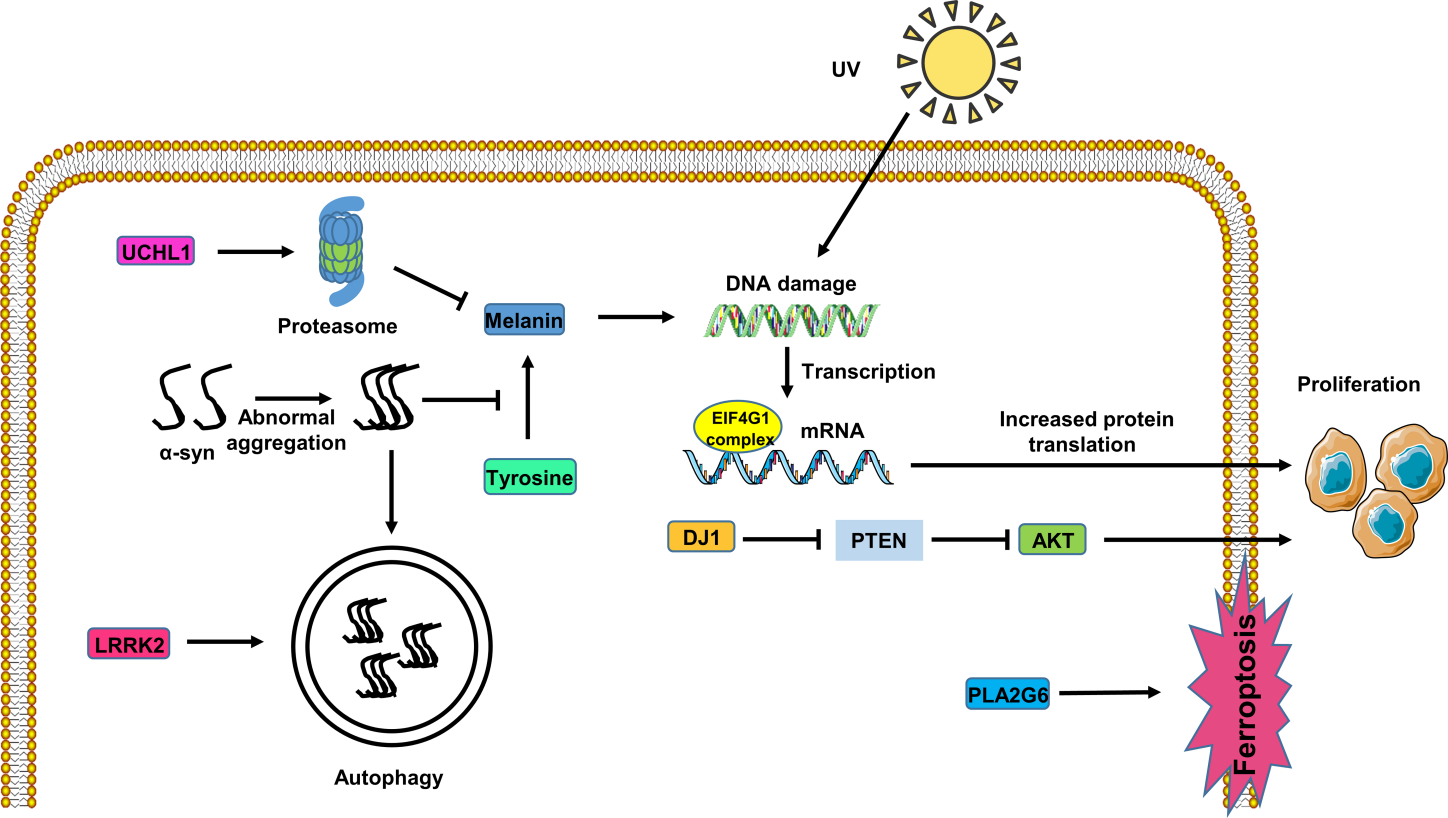
Potential role of the *PARK* genes in melanoma cells. *UHCL1* expression activates the proteasome pathway, inhibiting melanin synthesis. Abnormal accumulation of α-syn inhibits melanin synthesis, rendering DNA susceptible to damage from UV radiation in melanoma cells. *LRRK2* affects α-syn degradation through the autophagy pathway in melanoma cells. *PLA2G6* expression inhibits melanoma cell ferroptosis. The EIF4F complex alters the initiation of mRNA translation, promoting melanoma cell proliferation. DJ1 expression fosters melanoma cell proliferation through the PTEN/AKT pathway.

PD exhibits significant clinical and genetic diversity. While its intricate causes and pathological mechanisms have hindered breakthroughs in disease-modifying therapies, recent genetic technologies have advanced research approaches. In this context, mitochondrial dysfunction has been recognized as a central pathogenic factor in both familial and sporadic PD cases. *PARK* genes play a pivotal role in maintaining mitochondrial homeostasis, overseeing processes including biogenesis and mitophagy, as well as functions such as energy production and oxidative stress regulation. These genes can interact with the autophagy pathway, initiate proinflammatory immune responses, and exacerbate oxidative stress, all of which contribute to the aggregation of α-synuclein. Thus, rectifying mitochondrial dysfunction emerges as a promising therapeutic approach for neuroprotection in PD, targeting the underlying mechanisms that lead to neuronal damage. Additionally, the SNCA gene, which encodes α-synuclein and is alternatively known as *PARK1* or *PARK4*, is a significant causative factor in PD. Under normal physiological circumstances, α-synuclein may participate in functions like the preservation of synaptic structures and the facilitation of neural plasticity (22). Accumulation of misfolded α-syn in the brain induces the death of dopaminergic neurons in patients with PD (23). Notably, phosphorylated α-syn was detected in peripheral tissues of patients with PD especially at serine-129, which is the key event responsible for the formation of Lewy bodies in PD (24, 25). Tyr, an oxidase, serves as the rate-limiting enzyme in melanogenesis, while TH governs dopamine synthesis. α-syn interacts with Tyr, inhibits TH activity, and impedes dopamine and melanin synthesis (26, 27). Pan et al. demonstrated that α-syn overexpression in A375 melanoma cells reduces UV irradiation-induced melanin synthesis (26). This suggests that α-syn disrupts melanin production, which may enhance UV-induced DNA damage and, consequently, promote melanoma development (**Figure 1**). Furthermore, the *PMEL* gene encodes, a scaffold protein for melanin polymerization within melanosomes, and interacts with α-syn (28, 29), disrupting enzymes involved in melanin biosynthesis.

A previous study revealed aggregation of α-syn in dermal nerve fibers and melanomas from patients with PD (30, 31). Conversely, healthy melanocytes do not exhibit detectable levels of α-syn (32), implying its specific aggregation in individuals with PD having afflicted skin. Interestingly, trace amounts of α-syn have been identified in the skin of patients with melanoma (33), indicating that α-syn is a nexus linking PD and melanoma. Moreover, the knockdown of α-syn expression inhibits invasion and migration by SK-MEL-28 and SK-MEL-29 melanoma cell lines. Gajendran N, Rajasekaran S, et al., used two human melanoma cell lines (SK-MEL-28 and SK-MEL-29), SNCA gene knockout (KO) clones, and two human SH-SY5Y neuroblastoma cell lines. In the melanoma cell lines, the absence of α-synuclein expression led to a significant decrease in the expression of L1 cell adhesion molecule (L1CAM) and N-cadherin, and also significantly weakened cell motility. Compared with the control group, the motility of the four tested SNCA-KO cells was reduced by an average of 75% (34). Turriani E, Lázaro DF, et al., found that particularly in advanced melanoma stages, the accumulation of α-syn ensures that autophagy is maintained at a homeostatic level, thereby promoting melanoma cell survival. In this experiment, treating melanoma cells with high α-synuclein expression with oligomer modulators that affect α-synuclein led to obvious changes in the morphology of melanoma cells and inhibited their proliferation (35). Knockdown of α-syn in SK-MEL-28 melanoma cells induces intracellular iron ions accumulation, triggering ferroptosis (36). These findings collectively suggest that aggregation of α-syn in PD may act as a catalyst for melanoma development by modulating melanocyte autophagy, ferroptosis, and melanin synthesis.

### 2.2 *Parkin/PARK2* deficiency promotes melanoma

Parkin (Park2), an E3 ubiquitin ligase, is critical for maintaining mitochondrial function by regulating mitochondrial biogenesis and degradation. However, recent evidence, as demonstrated by Dimasuay, Kris Genelyn, et al., suggests that Parkin is involved in promoting inflammation (37). Parkin plays a crucial role in degrading abnormally folded proteins, particularly in mitophagy (38). Mutations in the Parkin gene (PRKN) disrupt autophagy and proteasome pathways, widely considered as key pathogenic mechanisms in patients with PD (39, 40).

In addition, Parkin additionally functions as a cell cycle inhibitor and driver of apoptosis in melanoma cells. Mutations involving Parkin inhibit its ubiquitination function, thereby promoting survival of melanoma cell. Levin L, Srour S, et al.’s *in vitro* analysis indicated that wild-type Parkin exerts a tumor-suppressive effect in melanoma development, leading to cell cycle arrest, reduced metabolic activity, and apoptosis. Potential Parkin substrates in melanoma were identified using mass spectrometry-based analysis, and a functional protein association network was generated. The activity of mutant Parkin was evaluated through protein structure modeling and examination of Parkin E3 ligase activity. The Parkin-E28K mutation impairs Parkin’s ubiquitination activity and abolishes its tumor-suppressive effect. In summary, analysis of genomic sequences and *in vitro* data suggests that Parkin is a potential link between melanoma and Parkinson’s disease (41). Re-expression of Parkin in melanoma cell lines inhibits cell proliferation, whereas inhibition of Parkin in melanocytes stimulates cell proliferation (42). Parkin deficiency heightens cellular sensitivity to UV radiation and accelerates DNA damage (43). And overexpression of Parkin reduces melanoma cell growth and induces apoptosis (44). Nonetheless, Parkin plays a very important role in regulating melanoma cell proliferation, migration and resistance to UV radiation.

### 2.3 *UCHL1/PARK5* reduces melanin production in melanoma

The ubiquitin-proteasome system (UPS) plays a crucial role in numerous cellular processes, with UPS dysfunction correlating with pathological changes in PD. In the past ten years, scientists have uncovered that a cluster of seemingly unrelated neurodegenerative disorders—including Parkinson’s disease—share striking similarities in cellular and molecular biology. All these neurodegenerative conditions involve protein misfolding and aggregation, triggering the formation of inclusion body aggregates within cells. These aggregates often contain chaperone proteins and ubiquitin (the proteolytic signal for the 26S proteasome), which assist in refolding misfolded proteins. The identification of disease-causing gene mutations encoding multiple ubiquitin-proteasome pathway proteins in Parkinson’s disease has further solidified the link between the ubiquitin-proteasome system and neurodegeneration (45). Ubiquitin carboxy-terminal hydrolase L1 (*UCHL1*) belongs to the deubiquitinating enzyme (DUB) family and serves as a crucial regulator of free ubiquitin levels in neurons (46). A deficiency of *UCHL1* results in inadequate ubiquitination and subsequent protein accumulation in neurons (47). Dysregulation of UPS function is closely associated with abnormal α-syn aggregation. Previous studies have indicated decreased *UCHL1* expression in the substantia nigra region of patients with PD (48).

*UCHL1* overexpression in melanoma cells activates UPS-mediated degradation, consequently inhibiting microphthalmia-associated transcription factor (MITF) expression and reducing melanin production (49). This suggests a dual role for *UCHL1* in both PD and melanoma, emphasizing its significance as a potential therapeutic target in these conditions (**Figure 1**).

### 2.4 Role of *PINK1/PARK6* in melanoma

The *PINK1/PARK6* gene encodes a serine/threonine protein kinase localized in mitochondria, which is crucial for protecting cells against stress-induced mitochondrial dysfunction by promoting mitophagy (50). *PINK1* facilitates Parkin recruitment to mitochondria, promoting its ubiquitination and subsequent induction of mitophagy. Mutations in the *PINK1* gene are associated with early-onset PD (51). Mutations in PARK6 also have been found in PD patients (52).

In melanoma cells, the knockdown of PINK1 inhibits BAY 87-2243, a potent inhibitor of the first oxidative phosphorylation complex)-induced reactive oxygen species (ROS) accumulation, mitophagy, and cell death (53). In tumor tissues, the tumor suppressor PTEN induces expression of PINK1, while PINK1, in turn, regulates the PI3K/AKT signaling pathway (54). Phosphatase and tensin homolog (PTEN) is a tumor suppressor that regulates the PI3K/AKT signaling pathway and its mutation has been reported to frequently occur in many human cancer cells (55). The experimental results of Yoon Jin Lee et al. show that the expression of PTEN in melanoma is lower than that in normal skin. Therefore, the regulatory effect of PTEN on the PI3K/AKT pathway may inhibit the development of melanoma. This suggesting that PINK1 may also influence melanoma progression through this pathway (56). However, the precise mechanisms underlying the PINK1’s involvement in melanoma development necessitate further investigation.

### 2.5 *DJ1/PARK7* overexpression promotes melanoma

The protein DJ1, encoded by the PARK7 gene, is strongly associated with early-onset PD. DJ1 regulates intracellular redox balance, thereby inhibiting the accumulation of ROS and protecting dopaminergic neurons from α-syn aggregation-induced neurotoxicity (57). Mutations in PARK7 are associated with an early-onset familial form of PD (58). DJ1 is overexpressed in melanoma cells compared to healthy skin, which was found to reduce PTEN levels, thereby inhibiting the PI3K/AKT pathway and apoptosis in melanoma cells (56).

Additionally, the research results of Nerea Lago-Baameiro et al. show that PARK7-silenced uveal melanoma cells exhibit abnormalities in the PI3K/Akt pathway. In both primary and metastatic UM cell lines, a significant reduction in Akt phosphorylation is consistent with DJ-1 inhibition. The PI3K pathway is responsible for regulating cell survival, while the tumor suppressor gene PTEN antagonizes this pathway and is also inhibited by DJ-1. Therefore, DJ-1 overexpression not only promotes Akt phosphorylation but also enhances cell viability, indicating that DJ1 expression can promote the proliferation and invasion of uveal melanoma cells through the PTEN/AKT pathway (59) (**Figure 1**).

Moreover, a physical interaction between DJ1 and α-syn has been identified through molecular docking and protein–protein interaction network analyses. Modifying such interaction through drug administration may be a novel target for the treatment of melanoma. Quesnel A, Martin LD, et al. analyzed the expression profiles of The Cancer Genome Atlas (TCGA) extracted from the UCSC Xena database to determine the expression of α-synuclein and DJ-1 in primary and metastatic cutaneous melanoma (SKCM). Immunohistochemical techniques detected upregulated expression of aggregated α-synuclein in metastatic melanoma lymph nodes. Protein-protein interaction (PPI) studies showed that overexpression of α-synuclein in SK-MEL-28 cells promoted DJ-1 expression. Molecular docking analysis revealed that α-synuclein formed stable complexes with chemotherapeutic drugs such as temozolomide, dacarbazine, and doxorubicin, with differing binding modes. In temozolomide-treated SK-MEL-28 spheroids, the levels of both proteins decreased simultaneously, indicating that drug binding may affect protein-protein interactions and stability (60). These findings reveal the multifaceted role of DJ1 in both PD and melanoma, suggesting its potential as a therapeutic target in both conditions.

### 2.6 Role of *LRRK2/PARK8* in melanoma

Mutations in the Leucine-Rich Repeat Kinase 2 (*LRRK2*) gene represents one of the most prevalent genetic risk factors for PD (61). Mutations in *LRRK2* result in increased *LRRK2* kinase activity, which induces lysosomal dysfunction, accumulation of α-syn, and neuronal damage (62). Patients with PD may have hyperactivation of the *LRRK2* regardless of *LRRK2* gene mutations (63). Therefore, inhibition of *LRRK2* kinase and improvement of membrane transport and lysosomal function is a promising potential treatment for PD (64).

The connection between *LRRK2* mutations and melanoma development remains inconclusive (65). A previous study has reported an increased melanoma risk among patients with PD having *LRRK2* mutations (66). *LRRK2* is emerging as a critical therapeutic target for autosomal dominant Parkinson’s disease (PD). The primary genetic cause of familial PD, which constitutes roughly 5-6% of familial instances and 2% of sporadic cases, lies in mutations within the *LRRK2* gene. The most common mutation, G2019S, enhances kinase function, leading to phosphorylation of key serine sites that regulate *LRRK2* activity, such as Ser910 and Ser935, which contributes to PD development. Development of *LRRK2* inhibitors has become a focal area in PD therapy research. Preclinical studies have shown these inhibitors hold potential to alleviate PD-associated pathology by modifying the cellular distribution of *LRRK2* and decreasing phosphorylation. Beyond its kinase activity, *LRRK2* is implicated in autophagic processes and mitochondrial function. This involvement suggests that PD hallmarks like mitochondrial dysfunction and impaired autophagy could be tackled by *LRRK2*-targeted therapies. Additionally, selective *LRRK2* inhibitors demonstrate promise in PD treatment, and further exploration of *LRRK2*’s molecular role in PD is crucial for developing effective therapies that can enhance patient outcomes and mitigate disease progression (67).

Given *LRRK2*’s involvement in the autophagy pathway, it is proposed that *LRRK2* mutations in patients with PD impact α-syn clearance and aggregation, thereby influencing PD progression. Further exploration of this relationship is warranted to better comprehend the interplay between *LRRK2*, α-syn pathology, and melanoma development (**Figure 1**).

### 2.7 *HTRA2/PARK13* expression suppresses melanoma

*HTRA2*, a member of the serine protease family, plays a pivotal role in various physiological processes, including maintenance of mitochondrial homeostasis and regulation of apoptosis (68). Gialluisi et al. proposed PARK13 as a candidate gene for late-onset PD (69). Its significance in preserving mitochondrial function and its dysregulation in PD pathogenesis have been documented (70, 71). *PARK13* deficiency results in PD-like symptoms (72). Previous studies have reported that indirect phosphorylation of *HTRA2* by *PINK1* enhances cellular resistance to mitochondrial stress (70, 72).

Elevated expression of *HTRA2* promotes apoptosis and augments the sensitivity of uveal melanomas to radiation therapy. Livin, also called melanoma inhibitor of apoptosis protein, suppresses apoptosis by binding and inhibiting caspases 3, 7 and 9 (73). Overexpression of livin renders malignant melanoma cells resistant to apoptotic stimuli. Notably, cleaved livin, upon interaction with *HTRA2*, relinquishes its anti-apoptotic function and assumes pro-apoptotic effects in melanoma cells (74). Although Yan et al. demonstrated *HTRA2*’s capability to cleave livin *in vitro*, its necessity for livin cleavage in melanoma cells remains uncertain (75). These findings suggest a potential role for *HTRA2* in melanoma; however, the underlying mechanisms warrants further elucidation.

### 2.8 Knock down of *PLA2G6/PARK14* inhibits melanoma

*PLA2G6* encodes the iPLA2β protein, which participates in various physiological processes including lipid metabolism, maintenance of mitochondrial integrity, phospholipid remodeling, signal transduction and cell death (76, 77). Mutations in *PLA2G6* have been identified as significant contributors to PD (78, 79). Deficiency of *PLA2G6* promotes aggregation of α-syn, thus accelerating PD progression (80).

Moreover, *PLA2G6* plays an important role in melanoma. Genome-wide association studies have strongly linked the PLA2G gene with melanoma (81). In human melanoma tissues, *PLA2G6* expression is upregulated compared to adjacent tissues. Through the use of Oncomine and CCLE online databases, immunohistochemistry, RT-qPCR, and Western blot analysis, Yifei Wang et al. found that *PLA2G6* knockdown significantly inhibits melanoma cell proliferation and metastasis while promoting cell apoptosis (82). Interestingly, *PLA2G6* also mitigates ferroptosis in melanoma cells by regulating the transport of iron ions (82). Consequently, further exploration into the role of *PLA2G6* in melanoma deserves to be conducted to unveil its potential as a therapeutic target (**Figure 1**).

### 2.9 Role of *eIF4G/PARK18* in PD and melanoma

The *PARK18* gene functions as a crucial component of the translation initiation complex eukaryotic initiation factor 4F (eIF4F), which exhibits a significant association with the risk of developing PD (83, 84). However, eIF4G’s role as a PD gene remains somewhat contentious, given conflicting findings regarding the effects of eIF4G gene mutations on PD (85–87).

Conversely, studies have reported a higher prevalence of eIF4G mutations among melanoma patients (88). Mutations in eIF4G that perturb mRNA translation initiation may contribute to the proliferation of tumor cells (89), leading to drug resistance in melanoma (90). Targeting eIF4G and disrupting the EIF4F complex with the small molecule SBI-756 has shown promise in attenuating drug resistance in BRAF-mutant melanoma (91). Therefore, eIF4G emerges as a promising new potential target for therapeutic intervention in melanoma (**Figure 1**).

## 3 Discussion

This article provides an overview of the potential roles of PD-related genes (*PARK* gene family) in melanoma, including *PARK1*, *PARK2*, *PARK5*, *PARK6*, *PARK7*, *PARK8*, *PARK13*, *PARK14*, and *PARK18* (**Table 1**). Mutations in the PARK1 gene have been implicated in promoting the development of both PD and melanoma. α-syn encoded by the *PARK1* gene acts as a catalyst, promoting melanoma progression, which explains the increased risk of melanoma among individuals with PD. Consequently, drugs targeting α-syn accumulation may offer therapeutic potential in the treatment of melanoma. For example, in patients with more rapidly progressing PD, prasinezumab may reduce motor symptom progression to a greater extent (92). Syn-RIBOTAC was able to selectively degrade SNCA mRNA, which significantly reduces the level of α-syn (93). PD01A is in Phase II clinical trials and has a favorable safety profile (94). These drugs can promote the degradation of α-syn, or reduce the aggregation of α-syn, or inhibit the synthesis of α-syn, and are potentially valuable in the treatment of melanoma. Also, some drugs used to treat neurodegenerative diseases have potential therapeutic effects on melanoma (95).

**Table 1:** The potential roles of *PARK* genes in the regulation of PD and melanoma.

Symbol	Function in PD	Function in melanoma
*PARK1/4*	Abnormal accumulation of α-syn damages dopaminergic neurons.	Abnormal accumulation of α-syn affects melanin synthesis.
*PARK2*	Parkin is involved mitophagy and proteasome pathways.	Parkin deficiency inhibits apoptosis in melanoma cells.
*PARK5*	*UCHL1* is involved in proteasome pathways.	*UCHL1* overexpression activates the proteasome pathway to reduce melanogenesis.
*PARK6*	PINK1 is involved in mitophagy.	Unclear
*PARK7*	DJ regulates intracellular redox balance to inhibit ROS accumulation and protects dopaminergic neurons.	DJ1 promotes melanoma cell proliferation and invasion through the PTEN/AKT pathway.
*PARK8*	LRRK2 is involved in the autophagy.	LRRK2 may influence α-syn aggregation through autophagy.
*PARK13*	HTRA2 has an important role in maintaining mitochondrial function.	Unclear
*PARK14*	*PLA2G6* plays an important role in innermitochondrial membrane homeostasis.	*PLA2G6* affects melanoma cells proliferation through ferroptosis and apoptosis.
*PARK18*	Unclear	EIF4G1 gene mutations promote melanoma cell proliferation by affecting mRNA translation.

The *PARK2* gene exhibits divergent roles in PD and melanoma. In PD, *PARK2* protects neurons by promoting mitophagy through ubiquitination, whereas in melanoma, it acts as a cell cycle inhibitor and apoptosis driver. However, the role of *PARK2* in melanoma remains somewhat controversial, with evidence suggesting that its deficiency inhibits melanoma growth and metastasis (96).

*PARK7*, associated with early-onset PD, is significantly upregulated in melanoma, inhibiting apoptosis. Unlike its antioxidant function in PD, *PARK7* downregulates the PTEN-regulated PI3K/AKT pathway, thereby regulating melanoma cell proliferation. Furthermore, the interaction between *PARK7* and α-syn, although not well understood, may synergistically promote melanoma cell proliferation, given their elevated expression in melanoma.

*PARK6* facilitates mitophagy by recruiting *PARK2* to mitochondria. In melanoma, *PARK6* regulates proliferation through the PI3K/AKT pathways independent of the PINK1/Parkin pathway. Reduced *PARK14* expression promotes apoptosis in melanoma cells, and is implicated in ferroptosis due to its affect iron ion metabolism (82), suggesting its potential as a therapeutic target in melanoma.

Targeting *PARK18* plays an important role in combating drug resistance in melanoma. The precise function of *PARK13* in melanoma remains unclear, but it likely influences apoptosis and contributes to melanoma pathogenesis.

The correlation between *PARK* gene expression in melanoma and PD has not been fully elucidated. Previously, it was reported that approximately 48% of individuals carry at least one *PARK* gene mutation, while 25% had multiple *PARK* gene mutations in the melanoma tissue (41). *PARK1*, *PARK2*, *PARK5*, and *PARK7* are usually overexpressed in melanoma. *PARK1* expression in melanoma and PD contributes to disease progression. *PARK2*, *PARK5*, and *PARK7* expression promotes melanoma proliferation and migration, which are negatively correlated with PD.

In summary, elucidating the roles of *PARK* genes in melanoma is essential for understanding the disease pathogenesis and facilitating early diagnosis and treatment (**Figure 1**). Given the close relationship between PD caused by mutations in *PARK* genes and melanoma, special attention should be paid to melanoma development in individuals with early-onset PD. Clinically, early detection of melanoma in patients with PD is paramount, and regular dermatological surveillance, including skin biopsies, is recommended. Clinicians should also educate patients with PD regarding the risk of developing melanoma and encourage sun protection practices to prevent melanoma development in the early stages of PD.

## 4 Conclusion

Melanoma may manifest in individuals with early-stage PD, potentially impacting their quality of life and increasing the risk of mortality. Understanding the involvement of *PARK* family-associated genes in melanoma is essential for effectively managing both PD and melanoma. Close monitoring of patient’s skin condition during anti-PD medication treatment is imperative to optimize therapeutic approaches.
